# Pregnancy outcomes of women with mechanical heart valves

**DOI:** 10.1186/1749-8090-8-S1-O278

**Published:** 2013-09-11

**Authors:** A Kucuker, EGY Eyi, SA Kucuker, M Hidiroglu, O Yurdakok, Z Catav, A Kunt, E Sener

**Affiliations:** 1 Ataturk Education and Research Hospital, Cardiovascular Department, Ankara, Turkey; 2 Zekai Tahir Burak Gynecology and Obstetrics Hospital, High Risk Pregnancies Department, Ankara, Turkey; 3 Turkiye Yuksek Ihtisas Hospital, Cardiovascular Surgery Department, Ankara, Turkey; 4 Konya Training and Research Hospital, Cardiovascular Surgery Department, Konya, Turkey

## Background

Firm anticoagulation protocols do not exists for pregnant women with mechanical heart valve prosthesis (MHVP). Centers advise differing strategies ranging from termination of the pregnancies to LMWH use or continuation of the usual dose warfarin. This study investigates pregnancy outcomes at three different institutions.

## Methods

Sixty female patients who had MHVP during their childbearing ages were questioned about their gestational history following valve surgery and among them 21 patients had 30 pregnancies. Pregnancy and patient outcomes were recorded as having abortus, stillbirths, live births, embriopathies and valve thrombosis. The anticoagulation regiment used by each patient was recorded.

## Results

Ages of 21 patients with pregnancies ranged between 22-53. Outcomes of 30 pregnancies were as follows: 7 live births (6 healthy and one with warfarin embriopathy), 20 abortus in first trimester (16 medical and 4 spontaneous), 3 stillbirths. Two patients, both on LMWH, during their 2nd trimester required urgent operation with mechanical valve thrombosis, survived but ended-up with stillbirths. Among the patients who had healthy births, 3 had LMWH throughout the pregnancy, 3 had LMWH in the first trimester, warfarin between 12 and 36 weeks, then LMWH until delivery. Warfarin embriopathy occurred in one case where mother was using >5 mg during first trimester.

## Conclusion

This pilot study demonstrates surprisingly high rates of gestation among MHVP bearing women. Medical abortus seems to be the most frequent approach to pregnant women with MHVP. For women wishing to continue their pregnancy, anticoagulation regiment and outcomes are divers and inconclusive to suggest the superiority of one among others.

